# English vowel identification in quiet and noise: effects of listeners' native language background

**DOI:** 10.3389/fnins.2014.00305

**Published:** 2014-09-29

**Authors:** Su-Hyun Jin, Chang Liu

**Affiliations:** Department of Communication Sciences and Disorders, University of Texas at AustinAustin, TX, USA

**Keywords:** bilingual, native language, english vowel identification in noise, speech perception, masking release, multi-talker babble noise

## Abstract

**Purpose:** To investigate the effect of listener's native language (L1) and the types of noise on English vowel identification in noise.

**Method:** Identification of 12 English vowels was measured in quiet and in long-term speech-shaped noise and multi-talker babble (MTB) noise for English- (EN), Chinese- (CN) and Korean-native (KN) listeners at various signal-to-noise ratios (SNRs).

**Results:** Compared to non-native listeners, EN listeners performed significantly better in quiet and in noise. Vowel identification in long-term speech-shaped noise and in MTB noise was similar between CN and KN listeners. This is different from our previous study in which KN listeners performed better than CN listeners in *English sentence recognition* in MTB noise.

**Discussion:** Results from the current study suggest that depending on speech materials, the effect of non-native listeners' L1 on speech perception in noise may be different. That is, in the perception of speech materials with little linguistic cues like isolated vowels, the characteristics of non-native listener's native language may not play a significant role. On the other hand, in the perception of running speech in which listeners need to use more linguistic cues (e.g., acoustic-phonetic, semantic, and prosodic cues), the non-native listener's native language background might result in a different masking effect.

## Introduction

It is often challenging for listeners to recognize speech in the presence of background noise, especially when the speech signal is degraded or the listener is unfamiliar with the speech signal as well as the background noise (Jin and Liu, 2012). Generally, when speech stimuli are presented in noise, native listeners are more adept at understanding it compared to non-native listeners, which has been known as “native advantage” (Flege and Liu, 2001; Rogers et al., 2006; Cutler et al., 2008; Stuart et al., 2010; Van Engen, 2010).

Recently, Jin and Liu (2012) examined English sentence recognition of English-native (EN), Chinese-native (CN), and Korean-native (KN) listeners in two different types of noise, steady-state noise (LTSSN) and multi-talker babble (MTB) presented at various signal-to-noise ratios (SNRs). Typically, listeners benefit from momentary dips in amplitude-modulating noise such as MTB. As a result, listeners understand speech better in MTB than in steady-state noise, resulting in “release from masking” (Festen, 1993). Jin and Liu (2012) found that consistent with the previous studies, EN listeners performed better in all listening conditions, showing the “native advantage” and had greater masking release than did CN and KN listeners. More interestingly, they found that KN listeners showed significantly better performance than CN listeners in MTB even though no difference was found between the two non-native listener groups in sentence recognition *in quiet and in steady-state noise*. Based on such results, Jin and Liu (2012) hypothesized that listeners' speech perceptual strategy in noise might depend on the linguistic characteristics of their native language. For example, in a tonal language like Chinese, tonal changes are critical acoustic features and considerably affect sentence recognition for CN listeners (Fu et al., 1998). If noise is modulating in frequency and amplitude like MTB, it could interfere with CN listener's perception of tone variations in sentences. Therefore, compared to KN listeners whose native languages are not tonal (Jun, 2005), CN listeners might be less efficient at processing suprasegmental cues available in momentary dips of the MTB for sentence recognition. Another possibility was that KN listeners processed acoustic-phonetic cues of English speech sounds in MTB more efficiently than CN listeners. The goal of this study was to examine the later possibility regarding the processing of acoustic-phonetic cues for native and non-native listeners.

Previous studies on vowel perception in noise showed that the amount of native advantage was depending on SNR, noise type, and native English exposures. Cutler and her colleagues measured English vowel and consonant identification in MTB for English- and Dutch-native listeners (Cutler et al., 2004, 2005). The results showed that the native advantage was similar regardless of noise levels (e.g., quiet, 0, 8, and 16 dB SNR), suggesting that if the speech materials were simple and sub-lexical like consonants or vowels, the effect of noise was the same for native and non-native listeners. That is, there is no additional native advantage in vowel or consonant perception in noise compared to the advantage in quiet (for a review, see Garcia Lecumberri et al., 2010).

However, a recent study by Mi et al. (2013) showed that non-native listeners were affected by noise more than native listeners in vowel perception. They measured English vowel identification of EN and CN listeners in steady-state noise and MTB at SNRs from −15 to 0 dB, which were much more difficult listening conditions than what the previous studies used (Cutler et al., 2004, 2005). Mi et al. (2013) found that the amount of native advantage depended on the noise type and non-native listeners' English exposure. For example, the native advantage for Chinese listeners in the US became larger as SNRs increased for both steady-state noise and MTB, while the native advantage for Chinese listeners in China increased from low to medium SNRs and then declined at higher SNRs for MTB. Results of these previous studies suggest that vowel perception of non-native listeners were dependent on several factors such as speech materials (e.g., isolated vowels or vowels embedded in CV or VC syllables), noise types (e.g., steady-state noise, twelve-talker babble vs. six-talker babble), SNRs, and non-native listeners' English exposures. By far, studies of non-native listener's vowel perception in noise investigated the performance difference between EN listeners and non-EN listeners (e.g., EN and Dutch native listeners in Cutler et al., 2004) or the effect of English exposure for the non-native listeners whose L1 was controlled (e.g., Chinese-native listeners in the US VS those in China in Mi et al., 2013). There is still lack of study investigating the role of *non-native listener's* L1 in vowel identification in noise. The current study examined English vowel identification in noise for native and non-native listeners. Specifically, we examined English vowel perception in noise for CN and KN listeners who have been living in the US and compared their performance with the performance of EN listeners. The main purpose of the current study was to investigate the effect of listener's *native language (L1)* on English vowel identification in modulating noise. Presumably, for phonemic stimuli like vowels and consonants, listeners mainly rely on acoustic-phonetic cues rather than suprasegmental cues to identify phonemes. Thus, results of vowel identification in LTSSN and babble will reveal whether the difference in sentence recognition between CN and KN listeners (Jin and Liu, 2012) was associated with the difference in the processing of acoustic-phonetic cues between the two non-native groups, if any.

## Methods

### Listeners

Participating in the study were three groups of young listeners, EN, CN, and KN. All the listeners were 19 to 30 years of age and had normal hearing with pure-tone thresholds = 15 dB HL at octave intervals between 250 and 8000 Hz (ANSI, 2010). Each group consisted of 12 undergraduate and graduate students who enrolled at the University of Texas at Austin. The non-native listeners had received school-based English education in their middle and high schools starting from the ages of 11–12 years old. All of the non-native listeners had US residency of 1–2 years: the average US residency was 1.6 years (STD = 0.2) for CN listeners (5 males and 7 females) and 1.5 years (STD = 0.3) for KN listeners (5 males and 7 females). In addition, the non-native listeners had TOEFL (Test of English as a Foreign Language) scores of at least 213 (computer-based tests, Lee et al., 2005). The experimental procedure including human subject recruitment and their participation to the current study has been reviewed and approved by the Institutional Review Board at the University of Texas at Austin prior to data collection.

### Vowel stimuli and noise

Twelve American English vowels /æ, ε, e, i, I, ɑ, ɔ, o, ʌ, u, ʊ, ɜ/ were used as speech stimuli (Hillenbrand et al., 1995). Vowel stimuli were originally recorded in the syllable context of /hVd/ produced by a young female native speaker of American English. To isolate the central vowel nucleus, we removed the onset and offset formant transitions of each syllable. The duration of isolated vowels was equalized to 170 ms. Vowels were presented in quiet and in noise with six SNRs at −15, −12, −9, −6, −3, and 0 dB.

Two different types of noise, long-term speech-shaped noise, LTSSN (steady-state noise) and English MTB (modulating noise) were presented at 70 dB SPL. The steady-state noise was generated from Gaussian noise that was shaped by a filter with an average spectrum of the twelve-talker babble. The twelve-talker babble was generated by mixing speech recordings of a section from a child's storybook, recorded from two repetitions of three female and three male adult speakers (Kalikow et al., 1977). Vowel sounds were centrally placed within the 400-ms masker. For each trial in the babble conditions, a 400-ms segment was randomly selected from a 10-s recording of the twelve-talker babble. Vowel stimuli and maskers had 10-ms rise-fall ramps. For calibration purposes, each vowel stimulus was equalized to the same root-mean-square (RMS) level and a 3.4-s segment was then generated by concatenating 20 repetitions of the RMS-equalized vowel. The sound-pressure levels of the vowels and maskers were calibrated at the output of the insert earphones (Etymotic ER-2) via a G.R.A.S. IEC 126 2-cc coupler connected to a Larson-Davis sound-level meter (Model 2800).

### Procedure

Listeners were seated in a sound-treated booth and in their right ear received, via ER-2 insert earphones, vowel stimuli and noise, digitized at 12,207 Hz. Stimulus presentation was controlled by a series of TDT hardware modules including an enhanced real-time processor (RP2.1), programmable attenuator (PA5), signal mixer (SM5), and headphone buffer (HB7). Listeners were seated in front of an LCD monitor that displayed 12 response alternatives as a text box labeled with the /hVd/ context (e.g., had, hawed, hayed, head, heed, heard, hid, hod, hoed, hood, hud, and who'd) corresponding to each vowel. Listeners responded by using a computer mouse to click on the button corresponding to their response choice. After hearing each vowel presentation, listeners were required to respond within 10 s. Prior to data collection, each listener went through a 15-min training session which was designed for the participants to be familiarized with experimental procedure. During the training session, vowels spoken by two native male speakers were presented in a /hVd/ context and feedback was provided to indicate the correct response on each trial, while no feedback was provided during the test session.

Under each test condition (quiet or noise), vowel identification was measured in one block of 240 trials, in which each of the 12 vowels were presented, randomly, 20 times. Therefore, vowel identification score in percent correct was calculated based on the 20 judgments for each vowel and each condition. Short breaks were provided between blocks and all test conditions in vowel identification were completed in two sessions with each session lasting approximately 2 h. Upon the completion of the training session, each listener had vowel identification in quiet first, followed by the two masker conditions. For the two masker conditions, six of the listeners in each group started with LTSSN, while the other six started with MTB. For a given type of masker, the order of the six SNRs was randomized; however, the SNR was fixed for a given block. The procedure was executed using Sykofizx® software.

## Results

### Vowel identification in quiet for native and non-native listeners

The average vowel identification scores in quiet were highest among EN listeners (88%, standard error = 13%), lowest among KN listeners (58%, standard error = 17%) and in between for the CN listeners (63%, standard error = 21%). To minimize a possible ceiling and/or floor effect, the identification scores in percentage were converted to rationalized arcsine transformed units (RAU, Studebaker, 1985). To examine the significance of the main factors regarding vowel identification, the researchers conducted a two-way (between-subject factor: listener language group and within-subject factor: vowel category) ANOVA with the RAUs as a dependent variable. The results showed that vowel identification was significantly affected by the two factors [listener language group: *F*_(2, 33)_ = 23.16, *p* < 0.05, η^2^_*p*_ = 0.0.537; and vowel category: *F*_(11, 363)_ = 23.02, *p* < 0.05, η^2^_*p*_ = 0.526]. The interaction between the two factors was also significant [*F*_(22, 363)_ = 3.5, *p* < 0.05; 0.182]. Tukey *post hoc* tests suggested that vowel identification of native and non-native listener groups differed significantly (*p* < 0.05) whereas no significant difference was found between CN and KN listeners (*p* > 0.05). Additional analysis grouped the 12 vowels into three groups: front (/æ, ε, e, i, I/), central (/ʌ, ɜ/) and back (/ɑ, ɔ, o, u, ʊ/), based on the tongue position of vowel production. Based on the three groups, a Two-Way ANOVA (between-subject factor: listener language group, within-subject factor: vowel groups) was conducted. There were significant listener group effect [*F*_(2, 33)_ = 21.56, *p* < 0.05, η^2^_*p*_ = 0.566] and vowel group effect [*F*_(2, 66)_ = 39.08, *p* < 0.05, η^2^_*p*_ = 0.542]. The interaction between the two main factors was also significant [*F*_(4, 66)_ = 2.96, *p* < 0.05, η^2^_*p*_ = 0.152]. Tukey *post hoc* tests revealed that the identification of back vowels was significantly lower than that of front and central vowels (both *p* < 0.05) but the identification scores of front and central vowels were not significantly different (*p* > 0.05). Within each vowel group, there was significant difference in vowel identification between native and non-native listeners whereas there was no difference between CN and KN listeners.

### Vowel identification in noise for native and non-native listeners

**Figure 1:**
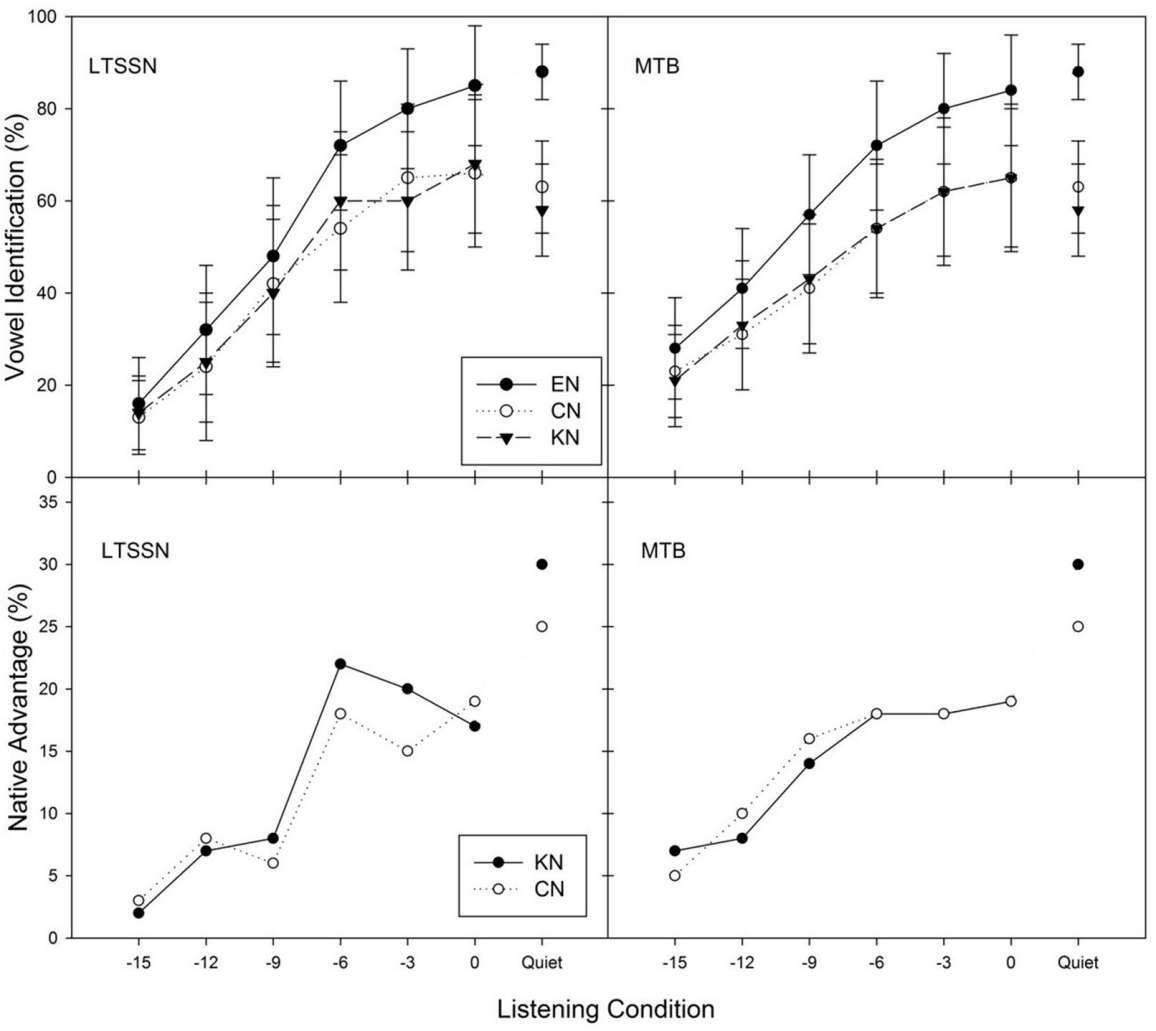
**Average percent correct vowel identification scores and standard errors (upper), and native advantage (lower) over the 12 vowels as a function of listening conditions in steady-state noise, LTSSN (left) and MTB (right) for three listener groups (EN, CN, and KN)**. Native advantage represents the difference in vowel identification scores between native and non-native listeners.

The upper panels of Figure 1 show, for the three listener groups, the average percent correct identification scores over 12 vowels as a function of SNRs in each noise condition (steady-state noise and MTB). Similar to vowel identification in quiet, EN listeners significantly outperformed CN and KN listener groups in every condition. In both noise conditions, however, no obvious difference separated CN and KN listeners. The native advantage, the performance difference between native listeners and non-native listeners, ranged from 3 to 22% in steady-state noise conditions and from 5 to 19% in MTB conditions, as illustrated in the lower panels of Figure 1.

The researchers conducted a four-factor (between-subject factor: listener group; within-subject factors: noise type, SNR, and vowel category) ANOVA with the RAU as the dependent variable. Significant effects were found for SNR [*F*_(5, 165)_ = 518.70, *p* < 0.05, η^2^_*p*_ = 0.94], vowel category [*F*_(11, 363)_ = 36.81, *p* < 0.05, η^2^_*p*_ = 0.527], listener group [*F*_(2, 33)_ = 16.46, *p* < 0.05, η^2^_*p*_ = 0.499], and noise type [*F*_(1, 33)_ = 10.88, *p* < 0.05, η^2^_*p*_ = 0.24]. Most of the two-factor and three-factor interaction effects were significant (all *p* < 0.05), aside from that of noise type x listener group [*F*_(2, 33)_ = 0.46, *p* > 0.05], noise type × SNR × listener group [*F*_(10, 165)_ = 1.15, *p* > 0.05] and noise type × vowel × listener group [*F*_(22, 363)_ = 1.29, *p* > 0.05]. The four-factor interaction was also not significant [*F*_(110, 1815)_ = 1.20, *p* > 0.05]. A Tukey *post hoc* test indicated that the EN listeners performed significantly better than the two non-native listener groups (all *p* < 0.05), while CN and KN listeners showed no significant difference in vowel identification in noise (*p* > 0.05).

In order to further examine whether vowel identification of each listener group might be affected by different vowel groups based on different tongue positions of vowel production, we also analyzed a four-factor (between-subject factor: listener group; within-subject factors: noise type, SNR, and vowel group) ANOVA. Significant effects were found for listener group [*F*_(2, 33)_ = 15.22, *p* < 0.05, η^2^_*p*_ = 0.479], vowel group [*F*_(2, 66)_ = 82.65, *p* < 0.05, η^2^_*p*_ = 0.714], noise type –[*F*_(1, 33)_ = 10.04, *p* < 0.05, η^2^_*p*_ = 0.233] and SNR [*F*_(5, 165)_ = 468.95, *p* < 0.05, η^2^_*p*_ = 0.934]. Tukey *post hoc* tests showed identification scores for each vowel group were significantly different from each other (all *p* < 0.05). Because the interaction between listener group and vowel group was significant, three separate Two-Way ANOVAs (between-subject factor: listener group; within-subject noise type and SNR) were conducted to examine whether there was a significant listener groups effect within each vowel group. For each vowel group (front, back and central), there were significant listener group effects (all *p* < 0.05) mainly due to substantial performance differences between native and non-native listeners. It is noteworthy that there was no significant difference between CN and KN listeners' identification of each vowel group in noise.

### Sensitivity parameter (d′) for native and non-native listeners

To minimize the effect of listener's response bias on the analysis of vowel identification, we also calculated the sensitivity measure (d′) which incorporates both “hit (identification rate)” and “false alarm” based on Signal Detection Theory (Macmillan and Creelman, 2005) for each experimental condition. The d′ was computed as the difference between the z-transform of hits and the z-transform of false alarms for each vowel and listening condition.

To analyze the effect of listener group and vowel category on the d′ in quiet condition, a two-factor (between-subjects factor: listener group × within-subjects factors: vowel category) ANOVA with d′ as the dependent variable was conducted for the quiet condition. Results showed that there was a significant effect of listener group [*F*_(2, 33)_ = 18.686, *p* < 0.05, η^2^_*p*_ = 0.531] and vowel category [*F*_(11, 363)_ = 74.153, *p* < 0.05, η^2^_*p*_ = 0.692] as well as the interaction [*F*_(22, 363)_ = 6.389, *p* < 0.05, η^2^_*p*_ = 0.279]. Tukey *post hoc* tests suggested that EN listeners had significantly higher d′ than CN and KN listeners (*p* < 0.05), while the two non-native groups had no difference (*p* > 0.05).

In addition, a four-factor (between-subjects factor: listener group × within-subjects factors: vowel category, SNR, and noise type) ANOVA with the d′ as the dependent variable was conducted for the noise conditions. Results showed that there was a significant effect of listener group [*F*_(2, 33)_ = 14.262, *p* < 0.05, η^2^_*p*_ = 0.464], vowel category [*F*_(11, 363)_ = 58.314, *p* < 0.05, η^2^_*p*_ = 0.639], and SNR [*F*_(5, 165)_ = 440.575, *p* < 0.05, η^2^_*p*_ = 0.930], but no significant effect of noise type [*F*_(1, 55)_ = 0.955, *p* > 0.05]. Interaction between SNR and listener group [*F*_(10, 165)_ = 9.305, *p* < 0.05, η^2^_*p*_ = 0.361], SNR and noise type [*F*_(5, 165)_ = 10.880, *p* < 0.05, η^2^_*p*_ = 0.248], and SNR and vowel category [*F*_(55, 1815)_ = 16.725, *p* < 0.05, η^2^_*p*_ = 0.336]. Tukey *post hoc* tests indicated that EN listeners had significantly greater d′ than CN and KN listeners (all *p* < 0.05), while there was no significant difference between the two groups of listeners (*p* > 0.05).

After categorizing 12 vowels into three vowel groups (front, central and back), we conducted a four-factor ANOVA with the d′ as the dependent variable. Results showed that there was a significant effect of listener group [*F*_(2, 33)_ = 12.945, *p* < 0.05, η^2^_*p*_ = 0.440], vowel group [*F*_(2, 66)_ = 60.108, *p* < 0.05, η^2^_*p*_ = 0.646], and SNR [*F*_(5, 165)_ = 460.167, *p* < 0.05, η^2^_*p*_ = 0.933], but no significant effect of noise type [*F*_(1, 33)_ = 0.497, *p* > 0.05]. Of the multi-factor interactions, only the two-factor interactions of SNR x noise type [*F*_(10, 165)_ = 9.670, *p* < 0.05, η^2^_*p*_ = 0.257] and SNR × vowel group [*F*_(10, 330)_ = 27.521, *p* < 0.05, η^2^_*p*_ = 0.454] and the three-factor interaction of SNR × vowel group × listener group [*F*_(20, 330)_ = 1.685, *p* < 0.05, η^2^_*p*_ = 0.093] were significant. Tukey *post hoc* tests suggested that EN listeners had significantly better d′ than their non-native peers at each of the three vowel groups (all *p* < 0.05), while no significant difference was found between the two non-native groups (all *p* > 0.05).

**Figure 2:**
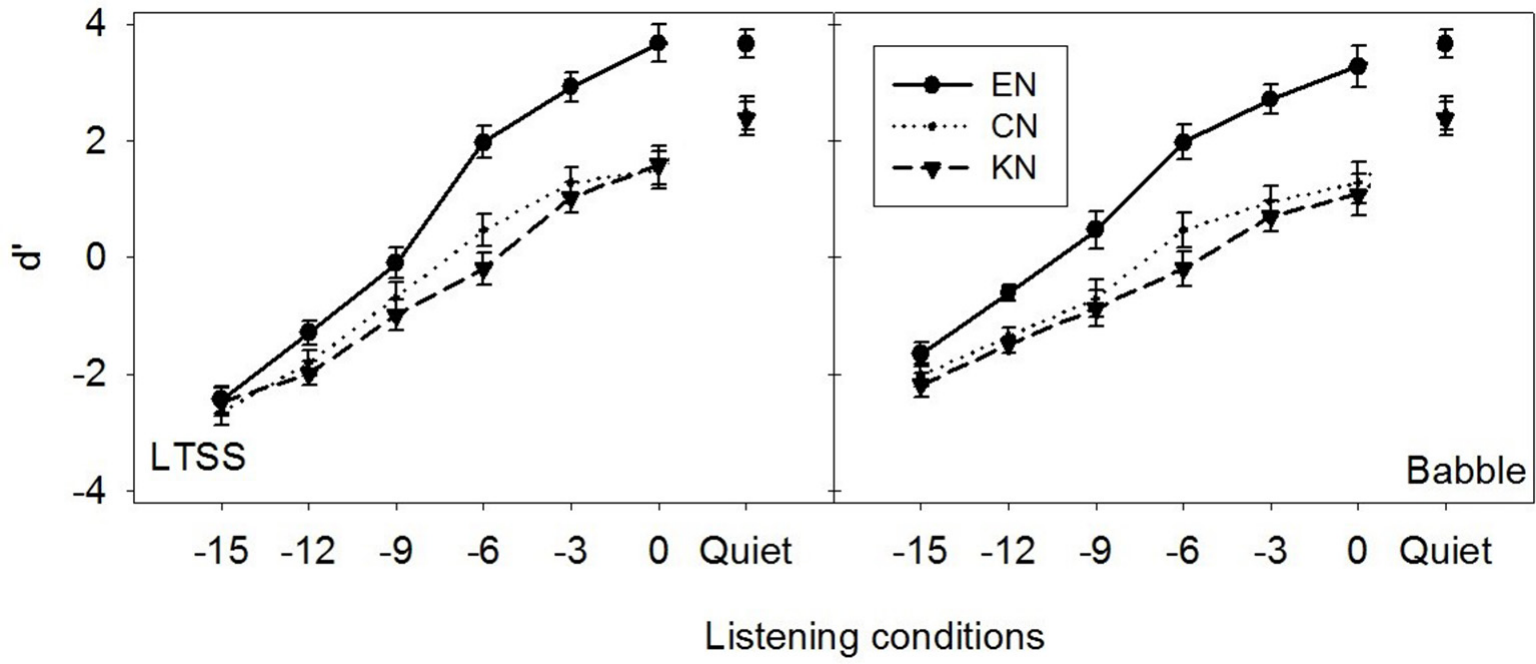
**Average d′ and standard errors over 12 vowels as a function of listening conditions in LTSSN (left) and MTB (right) for three listener groups (EN, CN and KN)**. The d′ was computed as the difference between the z-transform of hits and the z-transform of false alarm for each vowel and listening condition.

Overall, the statistical results on vowel identification scores and d′ indicated similar listener group effects: native listeners had significantly better vowel identification scores and d′ (e.g., vowel identifiability) than non-native listeners with no difference between the two non-native groups. However, the effect of noise type was significant only for vowel identification scores but not for the d′: vowel identification scores were significantly higher in MTB than in LTSS resulting in masking release, whereas the d′ in MTB and LTSS was quite similar as shown in Figure 2.

### Masking release for native and non-native listeners

**Figure 3:**
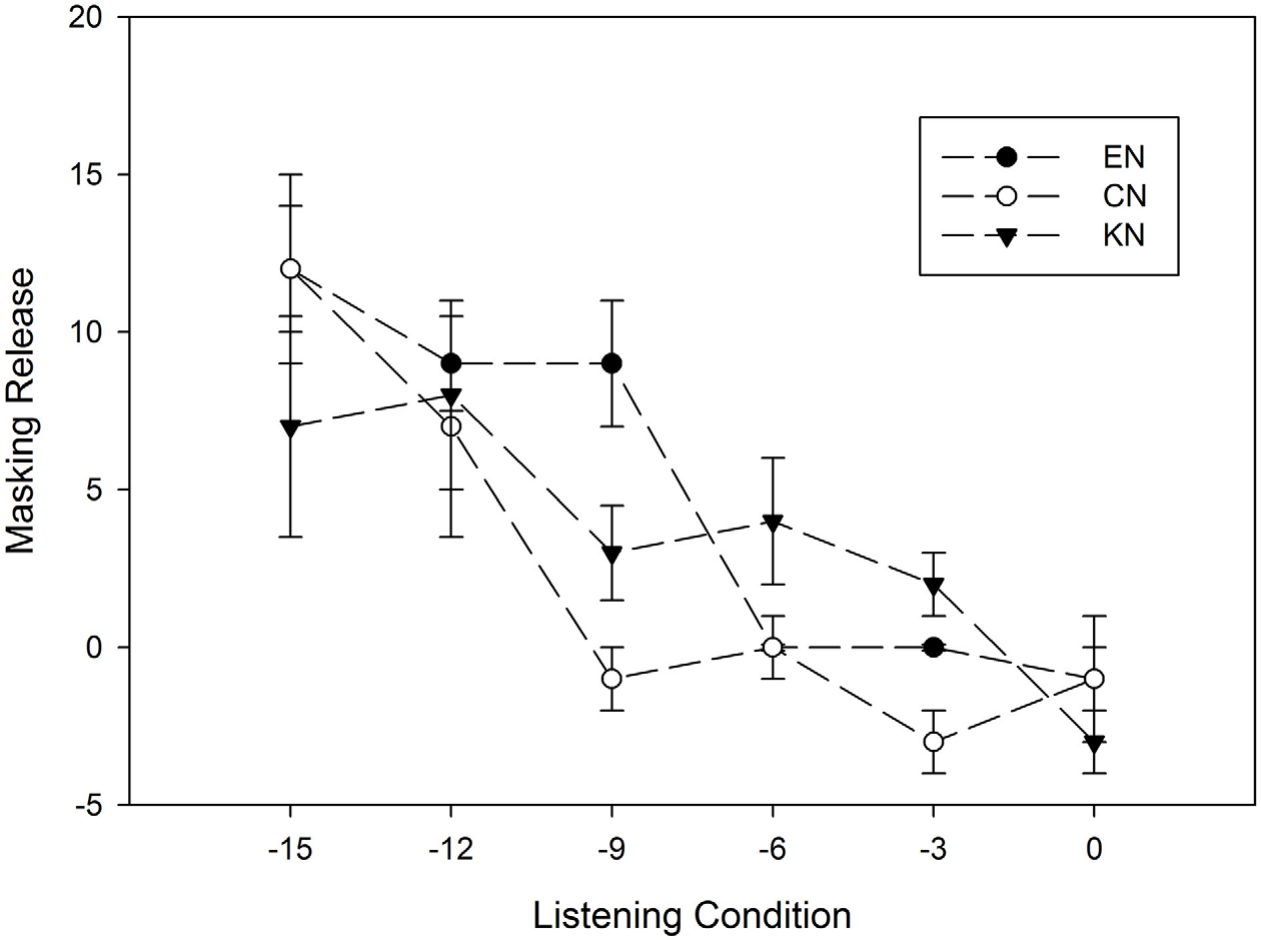
**The amount of masking release and standard errors as a function of SNRs for EN, CN and KN groups**. The masking release represents the difference between vowel identification score in MTB noise and those in steady-state noise (LTSSN). A positive masking release means better performance in MTB than in steady-state noise.

We examined the degree to which listeners benefited from the modulating noise over the steady noise, the difference between the vowel identification score in steady-state noise and that in MTB, at each SNR for the three listener groups. As Figure 3 illustrates, the EN group showed a higher masking release than both CN and KN groups at the lower SNRs (−15, −12, and −9 dB SNR). At the higher SNRs, the amount of masking release was similar for native listener group as it was for the non-native listener groups. A three-factor (between-subject factor: listener group; within-subject factors: vowel category and SNR) ANOVA with the masking release as the dependent variable showed significant effects of SNR [*F*_(5, 165)_ = 12.12, *p* < 0.05, η^2^_*p*_ = 0.71] and vowel category [*F*_(11, 451)_ = 5.14, *p* < 0.05, η^2^_*p*_ = 0.376]. However, there was no significant effect of listener group [*F*_(2, 33)_ = 0.6, *p* > 0.05, η^2^_*p*_ = 0.058]. Among the two- and three-factor interaction effects, only the interaction between vowel category and SNR was significant [*F*_(55, 1815)_ = 3.63, *p* < 0.05, η^2^_*p*_ = 0.51].

## Discussion

This study found no significant effect of non-native listener's L1 on English vowel identification in noise when the differences in the English-learning experience of non-native listeners were controlled. We carefully recruited Chinese and Korean listeners who had equivalent English education background, the age of arrival, the duration of residency in the US, and their English vowel identification performance in quiet. Chinese and Korean listeners showed comparable performances in all listening conditions including quiet, steady-state noise, and MTB. As shown in Figure 1, the vowel identification scores of Chinese listeners were similar to those of Korean listeners across noise type and SNRs. To evaluate listener's language effect on the identification of different vowel groups, 12 vowels were grouped into three categories, front, back and central, based on tongue positions of their production. All three groups of listeners showed the poorest identification scores for back vowels compared to those for front and central vowels. Within each vowel group, there was no significant difference in vowel identification scores between CN and KN listeners regardless of listening conditions. Furthermore, there was no significant group difference in the amount of masking release across the three listener groups (see Figure 3), which is consistent with the finding of Zhang et al. (2011) that EN and CN listeners showed equivalent masking release for word recognition in interrupted noise.

As shown in Figure 2, the d′ increased with SNR for all three groups and d′ was markedly higher for EN listeners than CN and KN listeners, who did not differ from each other. This is consistent with the results of vowel identification scores in percentage, suggesting that vowel identification was expectedly better for native listeners than for non-native listeners, but was similar between the two non-native groups. However, it should be noted that the effect of noise type was significant for vowel identification rate, but not for d′. As several previous studies of speech perception suggested (Broersma and Scharenborg, 2010; Jin and Liu, 2012; Mi et al., 2013), MTB contains temporal dips and provides the release of masking from the temporal variation, thus resulting in better perception performance than in stationary noise like LTSS noise. These results suggested that both native and non-native listeners were able to take the advantage of the temporal dips in MTB to identify vowels with higher identification accuracy, however, the temporal variation in MTB may also increase the rate of false alarms, which resulted in the similar d′ prime values to those in LTSS noise. In other words, the temporal dips of babble noise made vowel identification easier, but also increased the chance for false alarms, leading to similar vowel identifiability (e.g., d′) with LTSS noise.

These findings differ from earlier ones regarding sentence recognition in noise. A previous study of *English sentence recognition* in noise demonstrated that listener's L1 played a significant role to segregate sentence from modulating noise (Jin and Liu, 2012): Korean listeners understood English sentence significantly better than Chinese listeners in MTB despite there being no noticeable difference between the two listener groups in quiet and steady-state noise. Previous studies have reported that Chinese listeners were more affected by modulating noise (e.g., MTB or interrupted broad-band noise) and had smaller masking release in sentence recognition compared to EN listeners (Stuart et al., 2010; Van Engen, 2010) and KN listeners (Jin and Liu, 2012). On the other hand, perception of short stimuli like vowels or even words (Zhang et al., 2011) might not depend on non-native listener's native language background. Results from these studies suggest that the effect of non-native listeners' native language background on speech perception in noise may depend on *speech materials*. That is, when speech materials have limited cues such as vowels in which only acoustic-phonetic cues are available, the characteristics of non-native listener's native language may not play a significant role. On the other hand, for sentence recognition in which redundant cues are available (e.g., acoustic-phonetic, semantic, and prosodic cues), the non-native listener's native language background might result in a different masking effect (Cutler et al., 1992; Cutler and Otake, 1994; Jin and Liu, 2012). The lower English sentence recognition scores in MTB for CN listeners than for KN listeners were mainly due to the group difference in the processing of suprasegmental cues rather than acoustic-phonetic cues. It could be possible that because the current study used isolated vowels, the central vowel nuclei with equal duration of 170 ms, it might limit the application of the results to running speech. In particular, as Jin and Liu (2012) proposed that for sentence recognition, CN listeners may not be able to use the temporal dips of MTB or were affected by greater informational masking of MTB compared to KN listeners, the short duration of babble masker used in the present study (e.g., 400 ms) may minimize the differential effects of temporal dips and informational masking. To rule out such possibility, a future study is needed to examine the effect of listener's L1 on the recognition of CVC syllables or words that contain temporal and dynamic information as well as steady-state spectral information in noise.

Previous studies indicated that there is no additional native advantage in phoneme identification in noise (Cutler et al., 2004, 2005). Garcia Lecumberri and her colleagues argued that “noise has an equivalent overall effect” on phoneme recognition of native and non-native listeners (Garcia Lecumberri et al., 2010). However, the current study found that EN listeners identified vowels more accurately than CN and KN listeners regardless of the listening conditions and that the native advantage in vowel identification was largest in quiet and high-SNR conditions, decreasing as SNRs worsened, as shown in Figure 1 (bottom panels), which is also consistent with the finding from Mi et al.'s study (2013). The discrepancy in native advantage in vowel recognition in noise between the previous studies and the current one might be due to the different noise types and levels used by each study. The current study presented steady-state noise and 12 MTB noises at quite challenging noise levels, ranging from −15 to 0 dB SNRs while the previous studies (Cutler et al., 2004, 2005, for example) used six-talker babble noise at low noise levels such as 0, 8, and 16 dB SNRs. This suggests that when the listening condition is relatively easy (e.g., high SNRs), both native and non-native listeners might be equally affected by the noise in phoneme recognition, as shown the previous studies. On the other hand, in the very challenging listening environment, native listeners might still identify phonemes significantly better than non-native listeners, but such native advantages became smaller.

In fact, many studies in speech production of native and non-native speakers reported that speech intelligibility of non-native speakers was more negatively affected by noise than that of native speakers (Rogers et al., 2004; Wilson and Spaulding, 2010; Jin and Liu, 2013). Our recent study (Jin and Liu, 2013) suggested that noise background had significantly more impacts on vowel intelligibility of non-native speakers than on that of native speakers for medium SNRs (i.e., native advantage in vowel production was greatest at the medium SNRs, higher than low and high SNRs, and quiet). On the other hand, native advantage in vowel perception of this study was highest at high SNRs and quiet, showing a slightly different pattern from the native advantage in vowel production. The difference in the native-advantage pattern between vowel production and perception could be due to several possibilities: first, the non-native listeners in the two studies may differ in English proficiency, due to the different length of US residency (i.e., 1–5 years for the production study and 1–2 years for the perception study); second, the relationship between English vowel production and perception may not be so tight for non-native speakers than for native speakers. Perkell et al. indicate a close relationship between phonemic perception and production for native speakers (Perkell et al., 2004a,b), while such relationship for non-native speakers was not clear yet.

The current results showed that native listeners were able to use the acoustic-phonetic cues more efficiently than non-native listeners in quiet and higher SNRs. However, in sentence recognition, the native advantage was greatest at the middle SNR condition and less so at quiet, high-SNR, and low-SNR conditions (Jin and Liu, 2012). The difference in the native advantage between vowel identification and sentence recognition may result from the dissimilarity in available cues in vowels and sentences. As Mi et al. (2013) suggested, non-native listeners could, in hearing sentences, take advantage of redundant cues like acoustic, semantic, prosodic, and contextual ones such that the native advantage was reduced in quiet and high SNRs than in medium SNRs. On the other hand, non-native listeners have only limited acoustic-phonetic cues in isolated-vowel identification, resulting in their marked disadvantage compared to their native peers at high SNRs.

In summary, English speech perception of non-native listeners in noise might depend on several factors—speech materials, non-native listeners' language background, and the amount of English exposure. Listeners' native language (Chinese VS Korean) seemed to have a significant effect only on sentence recognition in noise (Jin and Liu, 2012) but not on vowel identification in noise. Due to limited access to speech cues in vowel identification, the level of native advantage was highest at lower SNRs. In contrast, for sentence recognition in noise, it was at its highest at middle SNRs (Jin and Liu, 2012). Although the effect of English learning on vowel identification in noise was not observed in the current study because of the inclusion criteria for non-native listeners, it could also be an important contributing factor as suggested by previous studies (Mi et al., 2013). Further studies are needed to examine English speech perception in noise with non-native listeners with different language background, speech materials (consonants or words), and different types of noise to generalize the current findings. In addition, studies of phonemic identification may need to analyze d′ prime values of identification performance taking a consideration of both false alarm rate and hit rate in order to better understand listener's recognition scores and perceptual strategy.

### Conflict of interest statement

The authors declare that the research was conducted in the absence of any commercial or financial relationships that could be construed as a potential conflict of interest.
